# Cost modelling rehabilitation in the home for reconditioning in the Australian context

**DOI:** 10.1186/s12913-023-10527-2

**Published:** 2024-01-30

**Authors:** Roslyn G. Poulos, Andrew M. D. Cole, Dan R. Hilvert, Kerry N. Warner, Steven G. Faux, Tuan-Anh Nguyen, Friedbert Kohler, Fey-Ching Un, Tara Alexander, Jacquelin T. Capell, Claire M. C. O’Connor, Christopher J. Poulos

**Affiliations:** 1 HammondCare, Sydney, Australia; 2 Hilvert Advisory, Sydney, Australia; 3 St Vincent’s Hospital, Sydney, Australia; 4 South Western Sydney Local Health District, Sydney, Australia; 5 Australasian Rehabilitation Outcomes Centre, University of Wollongong, Wollongong, Australia; 6 School of Psychology, UNSW, Sydney, Australia; 7 School of Population Health, UNSW, Sydney, Australia

**Keywords:** Rehabilitation, Rehabilitation in the home, Reconditioning, Delphi study, Allied health, Community rehabilitation, Post-acute rehabilitation, Cost analysis

## Abstract

**Background:**

Inpatient rehabilitation services are challenged by increasing demand. Where appropriate, a shift in service models towards more community-oriented approaches may improve efficiency. We aimed to estimate the hypothetical cost of delivering a consensus-based rehabilitation in the home (RITH) model as hospital substitution for patients requiring reconditioning following medical illness, surgery or treatment for cancer, compared to the cost of inpatient rehabilitation.

**Methods:**

Data were drawn from the following sources: the results of a Delphi survey with health professionals working in the field of rehabilitation in Australia; publicly available data and reports; and the expert opinion of the project team. Delphi survey data were analysed descriptively. The costing model was developed using assumptions based on the sources described above and was restricted to the Australian National Subacute and Non-Acute Patient Classification (AN-SNAP) classes 4AR1 to 4AR4, which comprise around 73% of all reconditioning episodes in Australia. RITH cost modelling estimates were compared to the known cost of inpatient rehabilitation. Where weighted averages are provided, these were determined based on the modelled number of inpatient reconditioning episodes per annum that might be substitutable by RITH.

**Results:**

The cost modelling estimated the weighted average cost of a RITH reconditioning episode (which mirrors an inpatient reconditioning episode in intensity and duration) for AN-SNAP classes 4AR1 to 4AR4, to be A$11,371, which is 28.1% less than the equivalent weighted average public inpatient cost (of A$15,820). This represents hypothetical savings of A$4,449 per RITH reconditioning substituted episode of care.

**Conclusions:**

The hypothetical cost of a model of RITH which would provide patients with as comprehensive a rehabilitation service as received in inpatient rehabilitation, has been determined. Findings suggest potential cost savings to the public hospital sector. Future research should focus on trials which compare actual clinical and cost outcomes of RITH for patients in the reconditioning impairment category, to inpatient rehabilitation.

**Supplementary Information:**

The online version contains supplementary material available at 10.1186/s12913-023-10527-2.

## Background

Rehabilitation for ‘reconditioning’ is defined as rehabilitation for ‘generalised deconditioning not attributable to any of the other impairment groups’ such as stroke, neurological conditions, and cardiac, orthopaedic or pain disorders (see Australasian Rehabilitation Outcomes Centre (AROC) impairment codes 16.1, 16.2 and 16.3 [1]). It is the largest (approximately 26% [2]) inpatient rehabilitation impairment type in Australia, and the number of episodes of reconditioning rehabilitation has doubled in a decade, increasing from 16,120 episodes in 2010 [3] to 32,877 in 2019 [4].

Inpatient rehabilitation units in both the public and private hospital sectors, are mostly working to capacity [5] and are challenged by increasing demand [6, 7]. One Australian study found that overall, patients requiring rehabilitation spent time equivalent to 12% of their acute hospital length of stay waiting for a rehabilitation bed [8]. Furthermore, the COVID-19 pandemic resulted in additional challenges for rehabilitation, including a reduction in available bed capacity and pressure for earlier patient discharge [9]. Rehabilitation patients may also be particularly vulnerable to COVID-19 due to their multiple comorbidities and/or older age, and opportunities for exposure and transmission of the virus occurring during therapy in common areas [10]. Thus, calls for shifting service models towards more community-oriented approaches are being made, both for improved efficiency and in the context of responding to COVID-19 [9].

Home-based rehabilitation as hospital substitution for stroke and orthopaedic conditions has been well described in the literature, with reports of comparable or better outcomes for appropriate patients compared to usual inpatient care (e.g., [11–15]). Further, systematic reviews of stroke trials [16, 17], and an individual trial of unilateral total hip or knee replacement [17] suggest that the costs are lower for home-based rehabilitation compared to usual inpatient rehabilitation. However, there is little research addressing rehabilitation in the home (RITH) for patients with generalised deconditioning, a group with substantial heterogeneity.

Thus, we sought to establish consensus on a model for RITH as hospital substitution for patients requiring reconditioning, through a three-round Delphi on-line survey with a multidisciplinary group of Australian health professionals working in the rehabilitation field. This research was undertaken during 2021/22, and was led by a multidisciplinary project team, consisting of academics (*n* = 4), rehabilitation physicians (*n* = 6), an occupational therapist (*n* = 1), and a health service financing consultant (*n* = 1). In summary, the first Delphi survey round was developed by the project team drawing on their combined expertise from working and researching in the rehabilitation setting, and informed by a rapid review of the literature on home-based rehabilitation services. Subsequent survey rounds explored issues for which consensus had not been achieved in a previous round, or tested issues that participants had raised in free text boxes. Survey methodology, individual survey items reaching consensus and the consensus model are reported in detail elsewhere [18]. Consensus was achieved on over 130 statements, leading to the development of a RITH for reconditioning model which consisted of five key steps aligned to the patient journey. The steps in the model were: initial patient identification; determination of eligibility and acceptance onto RITH; care plan development; program delivery; and discharge from RITH. Additional items related to the model covered clinical governance and budgetary considerations [18].

This paper estimates the hypothetical cost of providing the consensus-based model of RITH, where RITH mirrors an inpatient episode in intensity and duration. It also presents comparative cost estimates for RITH and inpatient rehabilitation for reconditioning and discusses the potential impact of RITH for reconditioning on aggregate public hospital rehabilitation expenditure.

## Methods

Cost modelling for RITH for reconditioning was undertaken utilising the following sources: Delphi survey consensus statements that related to the content or cost of a RITH program, and which have been reported previously [18]; publicly available data (population and inpatient rehabilitation statistics) and reports including Australian rehabilitation service standards and other documents that reported the content of inpatient rehabilitation and RITH for reconditioning or similar programs (referenced as appropriate in the Tables and text); and the expert opinion of the project team [18], who reviewed, discussed, and agreed upon the underlying model assumptions over the course of numerous research team meetings.

We also used the results of two questions which were developed for the third and final Delphi survey round to test the potential utilisation of RITH. A subset of the project team (*n* = 4) developed the questions, which were then tested and refined with the remainder (*n* = 8) of the team, before being included in the survey. The questions asked participants to use their clinical knowledge to estimate the potential utilisation of RITH for each of the six reconditioning case-mix classes used in Australia (Australian National Subacute and Non-Acute Patient classification (AN-SNAP) classes 4AR1 through 4AR6 [1]). Participants were asked two questions: ‘*What percentage of reconditioning patients in the following AN-SNAP classes do you think are likely to be suitable for RITH?*’ followed by *´What percentage of suitable reconditioning patients would you expect to actually want to take part in a RITH program if one were available?’*. Responses for each AN-SNAP class (4AR1 through 4AR6) were made using a slider which could be moved on a scale from zero to 100 percent. Participants who felt they had insufficient clinical familiarity with AN-SNAP classifications to answer the questions were asked to opt out from these, leaving only a subset of respondents.

### Data analysis

Descriptive analysis of Delphi data was undertaken using SPSS V27. Each Delphi participant’s estimated potential utilisation of RITH (by AN-SNAP class) was calculated from their estimate of the percentage of reconditioning patients suitable for RITH, multiplied by their estimate of the percentage of suitable reconditioning patients who might wish to take part in RITH if available. The median estimates for each class were determined.

Cost modelling was developed in MS Excel (Office 365) and was restricted to reconditioning classes 4AR1 to 4AR4. RITH program costs per episode were determined (Tables 3 and 4) and compared to the known inpatient cost per episode (Table 5). The assumptions and information sources underlying the calculations are detailed within each Table. Weighted averages for each metric in Tables 3, 4 and 5 were also determined, based on the modelled number of substitutable episodes per annum. The modelled number of substitutable episodes per annum is the number of inpatient reconditioning rehabilitation episodes that might be able to be substituted by RITH, limited to capital cities, and is based on the AN-SNAP reconditioning episodes reported to AROC in the 2019 (the last pre-Covid) year [2], adjusted for the potential utilisation of RITH determined from the Delphi survey (Table 2). The choice to limit the estimate to capital cities is conservative, since it assumes the operational efficiency available to RITH providers which arises from greater population density, may not be available elsewhere. Details of the calculations to determine the modelled number of substitutable episodes are shown in detail in Additional File 1.

## Results

### Delphi survey consensus statements relevant to the cost model

As previously reported, over 130 statements on aspects of the patient journey achieved consensus (that is, at least 70% of participants *agreed/strongly agreed*) during the Delphi survey [18]. The subset of these statements that were considered by the project team to have the most direct impact on the cost modelling for RITH are shown in Table 1. These statements relate to staff, program features and budgetary factors, and will be referred to in the text as T1.Item No.

**Table 1:** The subset of Delphi survey consensus statements that had the most direct impact on the cost model [18]

Category	Item No	Delphi statement
Staff	1	A case manager needs to be clinical
	2	A case manager should have administrative support
	3	A suitably skilled nurse/s should be part of a RITH team
	4	Allied health assistants have an important role to play in RITH
	5	Reconditioning following cancer should include psychosocial care delivered by a social worker and/or a psychologist
	6	If the carer is to partner in the patient’s rehabilitation (e.g. supporting therapy without a therapist present), then the RITH program must include time for carer education
	7	As long as team members know and understand their professional boundaries, an interdisciplinary approach can be an appropriate model of service provision for RITH for reconditioning
	8	The rehabilitation medicine physician should have a central role in the provision of RITH, as they do in inpatient rehabilitation units
Program features	9	Admission to inpatient rehabilitation should be available to RITH patients where progress has failed, and inpatient rehabilitation may assist
	10	RITH programs should not accept medically unstable patients
	11	The patient’s RITH care plan should include an indicative number and type of therapy interventions
	12	An acceptable key performance indicator (KPI) for subsequent admission to inpatient rehabilitation following a ‘failed’ RITH for reconditioning program is ≤ 10%
	13	In a well-functioning RITH program, acute hospital readmission rates should be as low as or lower than acute hospital readmission rates following inpatient rehabilitation
	14	Multi-disciplinary team case conferences should feature in each patient’s RITH program
	15	RITH patients should receive as comprehensive a rehabilitation service as they would have received if they had been undergoing inpatient rehabilitation
	16	Technology can be an effective means for a rehabilitation physician to monitor a patient’s progress during RITH
Budgetary features	17	The cost of a patient’s individual RITH program should be no more than the cost of a comparable inpatient rehabilitation episode
	18	A RITH service could use an external brokerage model to provide personal care, home help and meals when required by patients while they undergo RITH
	19	When required, paid support services (e.g. personal care, home help, meal services) should be available to patients on RITH programs, irrespective of whether they have a carer or not

### Estimated potential utilisation of RITH for reconditioning from the Delphi survey

Twenty-one participants (of 78 participating in the third Delphi survey round) indicated clinical familiarity with the AN-SNAP classification and responded to the questions relating to potential RITH utilisation. These were rehabilitation medicine physicians (*n* = 9, 42.9%), rehabilitation nurses (*n* = 6, 28.6%), physiotherapists (*n* = 3, 14.3%) and occupational therapists (*n* = 3, 14.3%). They estimated that around half of all patients in the higher functioning classes (AN-SNAP classes 4AR1 and 4AR2) may utilise RITH if it were available, but that few patients in the lowest functioning classes (AN-SNAP classes 4AR5 and 4AR6) would likely do so. See Table 2.

**Table 2:** Estimated potential utilisation of RITH by AN-SNAP class (*n* = 21 participants)

AN-SNAP (Version 4) class^a^	Median % of patients in class	25th -75th percentile
4AR1	49.7	27.8—78.5
4AR2	51.8	22.2—62.0
4AR3	20.7	7.9—41.5
4AR4	21.0	7.7—43.5
4AR5	7.2	1.0—23.6
4AR6	0.95	0.0—11.2

^a^Activity-based funding for admitted subacute care services in public hospitals is determined using the Australian National Subacute and Non-Acute Patient classification (AN-SNAP). The code 4AR is the general code that refers to patients requiring reconditioning. An AN-SNAP reconditioning class is assigned to a patient on admission to a rehabilitation program. The AN-SNAP reconditioning class is based on patient function, as follows [4]:

4AR1 Reconditioning, weighted Functional Independence Measure (FIM) motor 67‐91

4AR2 Reconditioning, weighted FIM motor 50‐66, FIM cognition 26‐35

4AR3 Reconditioning, weighted FIM motor 50‐66, FIM cognition 5‐ 25

4AR4 Reconditioning, weighted FIM motor 34‐49, FIM cognition 31‐35

4AR5 Reconditioning, weighted FIM motor 34‐49, FIM cognition 5‐ 30

4AR6 Reconditioning, weighted FIM motor 19–33

### Costing the RITH episode

The underlying assumption was that RITH should provide as comprehensive a rehabilitation service as would be provided by inpatient rehabilitation and be no more costly than inpatient rehabilitation (T1.15; T1.17). Cost modelling was restricted to the provision of RITH for patients in AN-SNAP classes 4AR1 to 4AR4 (which comprise around 73% of all inpatient reconditioning episodes in Australia [4]), as these were groups for whom Delphi survey participants estimated the potential utilisation would be greatest (Table 2).

#### Staff input

Table 3 shows the estimated staff input (occasions of service (OOS) per episode) required for RITH for reconditioning, drawing on T1.1 – T1.8 and T1.11. The specific information sources and assumptions underlying the estimates are shown in the right-hand column of Table 3. For allied health, clinical case manager, and rehabilitation nurse, the project team deemed each OOS as being 60 min with the patient, plus 30 min of preparation and documentation time, plus 40 min of travel time (130 min in total per OOS). On a weekly basis, this translates to:

- Twelve allied health OOS per week;
- Two clinical case manager *therapeutic* OOS per week. The clinical case manager could be either an allied health practitioner or a rehabilitation nurse (depending on patient need); and
- One rehabilitation nurse OOS per week.

**Table 3:** Estimated staff input for RITH for reconditioning programs

Occasions Of Service (“OOS”) and other clinical events per RITH reconditioning episode						
	**OOS per Episode by AN-SNAP Class**					**Assumptions and Information Sources**
	**4AR1**	**4AR2**	**4AR3**	**4AR4**	**Weighted Average**^a^	
**Allied health staff**						
Physiotherapist	6.0	7.3	7.8	9.5	7.0	- Allied Health staff mix and allied health OOS are derived from: the consensus view of the clinical co-investigators; the AFRM Inpatient Standards (2019) [19] and the AFRM Ambulatory Standards (2014) [20] for the Reconditioning impairment type; and allied health staff type by impairment group for reconditioning reported in the AROC Ambulatory Report 2021 [21] - From the above sources, we have derived and assumed percentage of allied health time as: Physiotherapist (30%); Allied Health Assistant (AHA) (20%); Occupational Therapist (20%); Exercise Physiologist (20%); Dietician (10%). Changes in allied health staff mix percentages, except for the proportion of allied health assistant input, will not materially impact the cost of providing RITH (shown in Tables 4 and 5) as allied health staff are costed the same, except for AHA’s who are costed less - From the above sources, two allied health sessions per day, for 6 of 7 days per week (with the weekend day being a Saturday) are assumed, resulting in an average of 1.7 allied health sessions per day over 7 days - We have assumed that each allied health OOS is 60 min with the patient, plus 30 min to allow visit preparation and documentation, plus 40 min of travel time (total staff resource investment of 130 min per OOS) - We have assumed that if a patient required social work or psychology input, these could be substituted for other clinical sessions - We have costed all allied health occasions of service as face-to-face, although it may be possible that some could occur via telerehabilitation - Based on the consensus view of the clinical co-investigators, an average of one weekly rehabilitation physician review (which could be by telehealth), is assumed, of 45 min duration plus 30 min preparation, documentation, and medical correspondence time (total of 75 min per OOS). An initial rehabilitation physician assessment in the acute hospital is not costed as we consider this predates the commencement of RITH - Published sources to assist in the determination of nursing and clinical case management OOS in RITH are limited. Based on the consensus view of the clinical co-investigators, an average of one OOS per week of nursing and two OOS per week of clinical case management is assumed, with each OOS comprising 60 min with the patient plus 30 min preparation and documentation time plus 40 min travel (i.e., 130 min per OOS) - It is recognised that some RITH for reconditioning patients might require greater nursing support, in which case a rehabilitation nurse can be designated the clinical case manager. There will be no material variation in the cost of RITH if the clinical case manager is a rehabilitation nurse or an allied health professional as both staff are costed at the same hourly rate - Based on the consensus view of the clinical co-investigators, allowance is made for one case conference per week for five clinical staff, including the rehabilitation physician; and daily MDT huddles on other weekdays. The duration of a case conference is assumed to be 15 min; the MDT huddles during the week are considered to be cost equivalent to one case conference - Based on the consensus view of the clinical co-investigators, allowance is made for two ‘planning/case coordination events’ per week to account for such things as rostering of staff, liaison with patients regarding appointments, organising case conferences, and other ad hoc administrative and reporting tasks to support RITH, communicating with patients, carers and family members about progress, liaising with and organising in-home community support services and arranging equipment. Each planning event is costed at 120 min of clinical staff time
Allied Health Assistant (AHA)	4.0	4.8	5.2	6.3	4.6	
Occupational Therapist	4.0	4.8	5.2	6.3	4.6	
Exercise physiologist	4.0	4.8	5.2	6.3	4.6	
Dietician	2.0	2.4	2.6	3.2	2.3	
* Allied Health OOS per episode*	*20.1*	*24.2*	*26.1*	*31.7*	*23.2*	
* Allied Health OOS per day*	*1.7*	*1.7*	*1.7*	*1.7*	*1.7*	
**Other clinical staff**						
Rehabilitation Physician	1.7	2.0	2.2	2.6	1.9	
Registered Nurse/Rehabilitation nurse	1.7	2.0	2.2	2.6	1.9	
Clinical case manager	3.3	4.0	4.3	5.3	3.9	
*Other Clinical OOS per episode*	*6.7*	*8.1*	*8.7*	*10.6*	*7.7*	
**Total clinical staff**						
*Total Clinical OOS per episode*^b^	*26.7*	*32.2*	*34.7*	*42.3*	*31.0*	
*Total Clinical OOS per day*^c^	*2.3*	*2.3*	*2.3*	*2.3*	*2.3*	
**Other clinical events**						
Case conferences/MDT huddle	3.3	4.0	4.3	5.3	3.9	
Clinicians per case conference/MDT huddle	5.0					
Planning/case coordination events per episode	3.3	4.0	4.3	5.3	3.9	
*Average Length of Stay (ALOS) per episode*^d^	*11.7*	*14.1*	*15.2*	*18.5*	*13.6*	

^a^This shows weighted averages for each metric where the weighting is based on the modelled number of substitutable episodes per annum (shown in Table 5, and calculated in Additional File 1)

^b^This is the sum of allied health OOS and other clinical OOS

^c^This is total OOS divided by average episode ALOS

^d^ALOS per AN-SNAP class is based on IHACPA Table 2021–22 [22]

For the rehabilitation physician,

- One OOS (via telehealth, T1.16) is provided per week. Each OOS was deemed to be 45 min, plus an additional 30 min of preparation, documentation and medical correspondence time (75 min in total). No junior medical officer support for RITH has been assumed in this cost model.

There is also allowance for:

- Two clinical case manager planning sessions per week. For consistency, each planning session is termed an OOS and was deemed to be 120 min.
- One case conference (T1.14) per week with five staff, and daily multi-disciplinary team (MDT) huddles (quick meetings which focus on each patient’s progression towards discharge) [23] as detailed in Table 3.

#### RITH program costs per episode

Table 4 shows the associated cost (in Australian dollars) of staff and other inputs required for RITH reconditioning episodes, along with the specific information sources and assumptions underlying the estimates presented. RITH program costs per episode are shown both by clinician type and by service type. Additional program costs include travel reimbursement (for staff use of own vehicles), home support services (e.g., meals, personal care assistance, T1.18, T1.19), and equipment depreciation (assuming an equipment loan pool for RITH patients of A$200,000).

**Table 4:** RITH program costs per episode (A$)

All costs in A$	**Cost per Episode by AN-SNAP Class**						**Assumptions and Information Sources**
	**4AR1**	**4AR2**	**4AR3**	**4AR4**	**Weighted Average**^a^	**Percent of modelled RITH cost**	
**Costs by clinician type**^1^							^1^ Clinical staff costings are based on mid-range experience level staff, and full-time equivalent annual salaries, based on NSW Health published award rates 2021 [24]. Hourly rates are then developed, adjusted for the following: - Staff are deemed only available to work 42 out of 52 weeks per year (the 10 weeks that staff are deemed to not be available are: 2 weeks of paid public holidays; 4 weeks of annual leave; 2 weeks of sick/carer’s leave; and 2 weeks to account for other leave types, such as paid parental leave and long service leave - We also assume that only 80% of clinical staff time is directly patient attributable. The 20% of time deemed not to be patient attributable is the time required for staff to attend to, for example, mandatory and other training, other administrative tasks and meetings - Hourly rates are based on a 38-h working week (or 7.6 h per day) - For allied health and nursing staff, hourly rates include apportioning the 50% Saturday salary loading - Hourly rates are inclusive of direct employment on-costs, which are superannuation entitlement at 10.5% of salary and workers compensation of 4.1% (workers compensation is based on NSW iCare premiums for 2020/21 for employees in the ‘Home Care’ category [25]) - Other (corporate) overheads are shown in Other (non-clinical) costs ^2^ Allied health clinicians costed at a weighted average of $96.08 per hour (allied health professionals [$104.43] and AHAs [$62.66]) ^3^ Rehabilitation nurses and clinical case managers are costed at $104.43 per hour ^4^ Rehabilitation physicians are costed at $227.15 ^5^ Based on the total clinical OOS shown in Table 3 ^6^ Based on 40 min of travel per in-home OOS ^7^ Based on one case conference [15 min] per week with 5 clinicians (incl 1 × Rehab Physician) plus 5 min per clinician per call for ‘tele-connectivity issues. The MDT huddles are considered cost equivalent to one case conference per week ^8^ Based on the clinical case manager spending 4 h (two 2-h planning/case coordination sessions [Table 3]) per week in planning and 2 h per week of clinical case manager patient service ^9^ An amount of $40 per episode (40 min per episode at a staff cost of $60/hour) is included to cover administrative support, including patient intake administrative tasks ^10^ A corporate overhead charge of 14.4% is used. This is based on a derivation from AIHW data for NSW public hospitals [26]. The derivation is calculated as follows: (administrative and clerical staff + other administrative expenses) / (total recurrent expenses, including depreciation) ^11^ Travel reimbursement to staff assumes staff using their private vehicles (i.e., not a fleet model for vehicles) and is based on 25 km of travel per in-home OOS, and a travel reimbursement to the staff member of 90 cents per km. The 25 km per in-home OOS travel assumes 40 min of travel at 37.5 km per hour ^12^ Based on $350 per week for in-home support services ^13^ Based on an average of $5 for consumables per in-home allied health or other clinical OOS ^14^ Annual depreciation over 5 years for an equipment pool of $200 k
Allied health clinicians^2^	$4,524	$5,452	$5,878	$7,154	$5,244	46.1	
Rehabilitation Nurse^3^	$410	$494	$532	$648	$475	4.2	
Rehabilitation Physician^4^	$728	$877	$945	$1,151	$844	7.4	
Clinical case manager^3^	$1,415	$1,705	$1,838	$2,237	$1,640	14.4	
Salary & on-costs per episode	$7,077	$8,528	$9,194	$11,190	$8,203	72.1	
**Costs by service type**							
Direct patient servicing time^5^	$2,712	$3,268	$3,523	$4,288	$3,143	27.6	
Travel time^6^	$1,626	$1,960	$2,113	$2,571	$1,885	16.6	
Case conferencing/MDT rapid rounds^7^	$685	$825	$889	$1,082	$794	7.0	
Case management & planning^8^	$2,054	$2,476	$2,669	$3,248	$2,381	20.9	
Salary & on-costs per episode	$7,077	$8,528	$9,914	$11,190	$8,203	72.1	
**Other (non-clinical) costs**							
Administrative support/intake^9^	$40	$40	$40	$40	$40	0.3	
Corporate overhead charge^10^	$1,410	$1,700	$1,832	$2,230	$1,635	14.4	
Travel reimbursement^11^	$564	$680	$733	$892	$654	5.8	
Home support costs^12^	$585	$705	$760	$925	$678	6.0	
Consumables^13^	$125	$151	$163	$198	$145	1.3	
Equipment depreciation^14^	$16	$16	$16	$16	$16	0.1	
Modelled RITH costs per episode	$9,817	$11,820	$12,737	$15,491	$11,371	100.0	

^a^This shows weighted averages for each metric where the weighting is based on the modelled number of substitutable episodes per annum (shown in Table 5 and calculated in Additional File 1)

The estimated hypothetical cost per RITH reconditioning episode ranges from A$9,817 (4AR1), to A$15,491 (4AR4), with a weighted average cost per episode of A$11,371. The weighted average RITH episode cost can be broken down into the following components:

- A$3,143 (27.6% of total) in direct patient servicing time costs;
- A$1,885 (16.6% of total) in staff travel time costs;
- A$794 (7.0% of total) in staff case conferencing and MDT huddle costs;
- A$2,381 (20.9% of total) in case management and planning costs;
- A$40 (0.3% of total) administrative support for client intake;
- A$1,634 (14.4% of total) in corporate overhead costs;
- A$654 (5.8% of total) in travel reimbursement costs;
- A$678 (6.0% of total) in-home support costs;
- A$145 (1.3% of total) in consumables costs;
- A$16 (0.1% of total) in equipment depreciation costs.

### RITH episode cost compared to that of inpatient rehabilitation

Table 5 estimates the potential cost savings per episode to the public hospital sector in Australia of the proposed RITH program compared to inpatient rehabilitation for reconditioning. The weighted average public inpatient cost per episode of A$15,820 has been estimated with reference to publicly available Independent Health and Aged Care Pricing Authority (IHACPA) data points. Each AN-SNAP class has an IHACPA associated cost weight (ranging from 2.3 for 4AR1 to 4.11 for 4AR4) ([22], p.84) and the IHACPA National Efficient Price per single cost weight of activity in 2021–22 was A$5,597 ([22], p.7). Thus, the derived price paid for an inpatient reconditioning episode in this period ranges from A$12,850 for 4AR1 to A$22,976 for 4AR4 (weighted average of A$15,820). The modelled RITH costs (calculations shown in Table 4 and reappearing in Table 5) indicate hypothetical savings per episode ranging from 23.6% for 4AR1, to 32.6% for 4AR4 (weighted average of 28.1%).

**Table 5:** Potential cost savings to the public hospital sector (A$)

*All costs in A$*	**Cost per Episode by AN-SNAP Class**					**Assumptions and Sources**
	**4AR1**	**4AR2**	**4AR3**	**4AR4**	**Weighted Average**^a^**/** **Total**	
**Cost savings to govt. per RITH episode**						^1^ IHACPA, National Efficient Price Determination 2021–22, Appendix 1 [22] ^2^ IHACPA, National Efficient Price Determination 2021–22, page 7 [22] ^3^ = Episodic cost weight x NEP per single cost weight ^4^ As shown in Table 4 ^5^ Current inpatient cost per episode LESS the modelled RITH episode cost ^6^ Cost savings attributable to RITH per episode expressed as a percentage of the current inpatient cost per episode ^7^ This is the number of inpatient reconditioning rehabilitation episodes that might be substitutable by RITH, limited to capital cities, and is based on the AN-SNAP reconditioning episodes reported to AROC in the 2019 (the last pre-Covid) year [2], adjusted for the DELPHI-derived anticipated utilisation of RITH (Additional File 1) ^8^ Potential annual cost savings to government from RITH if estimated substitutable episodes are taken up
Cost weight per episode type^1^	2.30x	3.01x	3.23x	4.11x	2.83x	
NEP per single cost weight^2^	$5,597					
Current inpatient cost per episode^3, b^	$12,850	$16,825	$18,064	$22,976	$15,820	
Less: modelled RITH costs per episode^4^	($9,817)	($11,820)	($12,737)	($15,491)	($11,371)	
*Equals: cost savings to govt. per episode*^*5*^	*$3,033*	*$5,005*	*$5,327*	*$7,486*	*$4,449*	
*Percentage cost savings to govt. per episode*^*6*^	*23.6%*	*29.7%*	*29.5%*	*32.6%*	*28.1%*	
**Potential cost savings to govt. per annum**						
Modelled number of substitutable episodes per annum^7^	881	1,205	258	115	2,459	
*Cost savings to govt. per annum*^*8*^	*$2.67 m*	*$6.03 m*	*$1.38 m*	*$0.86 m*	*$10.94 m*	

^a^This shows weighted averages for each metric where the weighting is based on the modelled number of substitutable episodes per annum (calculated as shown in Additional File 1)

^b^The National Efficient Price (NEP) is used to determine of the amount of Commonwealth Government funding for public hospital services and to provide a benchmark about the efficient cost of providing public hospital services [27]

### A conservative estimate of the potential impact of RITH for reconditioning on aggregate public hospital rehabilitation expenditure in Australia

A conservative estimate of potential cost savings to the Australian public hospital sector was quantified, using the modelled number of substitutable episodes per annum. Details on the derivation of the number of substitutable episodes are provided in Additional File 1. In summary, they relate to the provision of RITH in the capital city only [28] of each state or territory, and use the estimated potential utilisation of RITH for reconditioning for the four AN-SNAP classes 4AR1 through 4AR4 (Table 2), and the number of reconditioning episodes (4AR1 to 4AR4) reported to AROC in the 2019 year ([2], p. 92). Based on the above, an estimated 2,459 public inpatient reconditioning rehabilitation episodes could be substituted with a RITH program annually. The project team restricted the estimate to capital cities because the greater population density in these cities would allow RITH service providers operational efficiencies from having critical mass. This is a conservative approach since other large Australian cities may offer similar efficiencies. RITH in regional and rural locations, however, might require a greater allowance for staff travel costs.

Should 2,459 public inpatient reconditioning rehabilitation episodes be substituted with RITH, this would translate into hypothetical cost savings to government of A$10.9 million dollars annually (Table 5).

## Discussion

In this paper, costings of a consensus-based model for RITH as hospital substitution for patients requiring reconditioning (in AN-SNAP classes 4AR1 to 4AR4) have been presented, drawing on Delphi survey data [18], publicly available data, relevant reports and rehabilitation standards, and informed by the clinical and service experience of the research team. Costings have been applied to a RITH for reconditioning model which reflects an equivalent public hospital inpatient rehabilitation episode in terms of duration and intensity, with significant hypothetical cost savings per episode found for RITH when compared to inpatient rehabilitation.

### RITH intensity and cost

We have attempted to present a fully costed RITH hospital substitution program. While the actual amount of therapy intensity for inpatient rehabilitation is not formally reported, the Australasian Faculty of Rehabilitation Medicine (AFRM) Standards recommend that therapy should consist of a minimum of three hours per day over five days per week for patients who can tolerate it ([19], p.10). Published data suggest that actual therapy levels may be less, especially for older patients who are not presenting with a predominantly neurological impairment [29]. Our modelled allied health therapy intensity is an average of 1.7 h/day over 7 days, which is equivalent to an average of 2.4 h per day over five days. This does not include nursing input or clinical case management time, both of which are likely to include a therapy component.

Our costing model provides a total of allied health therapy per RITH episode that ranges from 20.1 (4AR1) through to 31.7 (4AR4) hours, which increases to a total of 26.7 h (4AR1) to 42.3 h (4AR4) with the inclusion of other direct clinical input (i.e., therapy plus clinical case management, plus nursing, plus rehabilitation physician time). Data from the AROC Ambulatory Report [21] indicate the mean number of occasions of service per episode for patients with a reconditioning impairment code in Australia in 2021 was 26.4 ([21], p.32). If we assume that an OOS is one hour (since it is not defined by AROC), then this is supportive of our model. Direct comparison is difficult however, because the AROC ambulatory dataset includes patients whose rehabilitation may have only been in an ambulatory setting, as well as those for whom it may have been a continuation of the inpatient rehabilitation episode; and the extent to which AROC-reported episodes were ‘hospital substitution’ is unknown. Interestingly, the mean length of program for the reconditioning impairment code in the Report is 62.8 days ([21], p. 26), which suggests therapy in the ambulatory setting was spread over a long duration. This would be more akin to an alternative RITH service delivery model which Delphi survey participants also supported [18] on the assumption that it would be of lower or varied intensity, delivered over a duration of up to ten weeks, and cost-equivalent to the intensive, shorter duration model costed in this paper.

Our modelled cost per episode of RITH for reconditioning ranges from A$9,817 (4AR1) to A$15,491 (4AR4), with the estimated weighted average cost being A$11,371. While we were unable to locate any publicly available data on the cost of other RITH programs for reconditioning as hospital substitution, some comparative data are available for other programs delivered in the home. The Transition Care Program (TCP) and the Short-Term Restorative Care (STRC) Program are Australian government funded programs targeting community-dwelling older people who have experienced functional decline and who have (in the case of TCP) and have not (in the case of STRC) experienced recent hospitalisation [30]. These programs offer short term support (8 – 12 weeks) with low intensity therapy aimed at improving function and reducing premature admission to residential aged care. The duration of the TCP and STRC programs are more akin to the alternative RITH program described above (that is, lower or varied therapy intensity of up to 10 weeks duration) and the AROC reported average duration of ambulatory rehabilitation for reconditioning ([21], p. 26).

The cost to government of delivering an STRC program, which runs over a maximum of 56 days, was set at A$214.39 per day for 2021–2022 [31], equating to just over A$12,000 per 56-day episode, which is similar to our weighted average cost per RITH episode. The reported cost of delivering the TCP program (up to 12-weeks) varies between jurisdictions, ranging from A$247.34 per day in the ACT to A$348.68 per day in Victoria (2018 financial year) [32]. This would equate to between A$20,000 and A$30,000 per 12-week episode, thus considerably higher than our weighted average cost per RITH episode. This may be somewhat accounted for by the fact that the cost of TCP appears to increase as the proportion of episodes delivered in residential care increases, while our RITH model assumes delivery in the home setting only (Table 4). Further, TCP may provide additional nursing support and personal care to that which we have allowed within our costing of RITH.

### Potential savings from RITH for reconditioning

We have shown hypothetical cost savings that range from 23.6% (4AR1) to 32.6% (4AR4) per episode (weighted average saving of 28.1%) compared to the cost of an inpatient rehabilitation program for reconditioning. Cost savings have been reported for existing RITH programs in other patient groups and for “non-rehabilitation” hospital-in-the home programs with partial or total episode substitution (e.g., [16, 17, 33–36]). For example, savings were found to range from 4%—30% for various stroke early supported discharge programs versus usual care [16], while an average saving of 26.5% [34] was reported in a meta-analysis of ‘hospital in the home’ studies, which suggest our estimated savings are credible.

The majority of our hypothetical cost savings of RITH for reconditioning over inpatient rehabilitation is likely accounted for by the absence of costs associated with 24/7 inpatient nursing care and hotel services. Counter to this saving is the requirement for staff to travel, which we estimated to be 22.4% of the cost of RITH (based on an allowance of 40 min travel per OOS plus a staff travel allowance of 90 cents per kilometre for use of their own vehicle, Table 4). Travel cost is a variable that will depend on several factors, comprising the location serviced (including distances to be travelled, traffic congestion, road tolls and parking costs) and economies of scale arising with patient volume. For these reasons we modelled the potential costs (and cost savings) for capital cities only. Some allowance for the provision of in-home support for patients and carers has been made (6.0% of program cost), but this could be increased depending on assessed patient need, while still remaining below the cost of an inpatient rehabilitation episode.

With good patient selection, we expect re-admission rates to acute care from RITH to be similar to those that occur for inpatient rehabilitation (T1.10, T1.13). However, we expect some patients (less than 10%, T1.9, T1.12) who undergo RITH to subsequently require inpatient rehabilitation. While this might dilute the savings, it is likely that patients who failed to adequately progress in RITH would have required a longer than average inpatient rehabilitation episode had they remained in a hospital rehabilitation ward, but we have no data available to support this assertion.

### Impact on the patient and their household

When inpatient care is shifted to the home, informal carers are to a greater or lesser extent undertaking some of the care-work that would have previously been undertaken by paid staff [37]. As carers are integral to the success of RITH programs for many patients, carers must be fully informed, willing and supported [18, 37]. The provision of in-home support in our model was an attempt to reduce carer burden. There is a need for future research to measure costs associated with the carer role in order to understand fully the implications of RITH as hospital substitution [37]. Despite the potential cost and care-shift [37], patients and carers who had experienced RITH for neurological and orthopaedic conditions, found being at home beneficial for therapy and for the well-being of both the patient and their family [38]; thus for appropriate patients (and carers), RITH provides increased choice about care.

### Potential aggregate savings to the public hospital sector

Our finding of potential aggregate public hospital system savings of A$10.9 million are based on a number of assumptions, including the estimated potential utilisation of RITH for reconditioning and the relative cost of inpatient rehabilitation versus our modelled RITH cost. Any such savings should be regarded as theoretical and imprecise for several reasons. First, estimated potential utilisation is based on the experience of a self-selected subset of survey participants and cannot be assumed to be generalisable (e.g., factors impacting patient selection may vary in different socio-demographic communities). Second, the uptake of RITH by individual health services may not be complete, and will vary depending on local factors (e.g., economies of scale that can be achieved, workforce availability, and competing service priorities). Third, ‘savings’ will only be achieved if inpatient rehabilitation bed utilisation is reduced, allowing actual savings to be realised.

In reality, ‘savings’ may better expressed as the delivery of greater capacity for the same overall system expenditure, leading to an alleviation of inpatient capacity constraints within the existing public hospital system. By extension, fewer additional inpatient beds may be required in the future, resulting in savings in both (a) capital expenditure (fewer beds needing to be built) and (b) recurring operational expenses (from the servicing of additional beds). Note that our modelling excludes any potential capital savings.

### Strengths and further limitations

A detailed discussion of the limitations of the Delphi survey methodology used has been presented elsewhere [18]. In addition to the limitations listed in the preceding section, there are several other limitations associated with our cost modelling of RITH episodes which must be acknowledged. Of note, our estimates are hypothetical only, since an operating program was not costed. Some of the assumptions used in our costings have relied on the clinical and service expertise of the authors, due to the absence of other information sources. While this could present a source of bias, assumptions have been made explicit and transparent through the details provided in Tables 3, 4 and 5 and Additional File 1. It is possible that additional costs may be incurred while the patient is at home, that have not been accounted for in the model, such as medical investigations, pathology, and non-routine medical assessments (i.e., beyond the rehabilitation physician reviews). However, as per our admission criteria for RITH [18], patients should enter RITH medically stable (T1.10). Conversely, there may be opportunities for trimming and/or reallocating costs based on individual service configurations and overhead costs. Medical staffing configurations may include the use of junior medical staff to defray the amount of medical consultant time required. The transparency of our assumptions should enable such modifications to be made by readers. We must also acknowledge that the cost model is vulnerable to unpredictable costs, such as higher readmission rates and poorer clinical outcomes (e.g., falls at home). On the other hand, calculated public hospital sector ‘savings’ have been limited to RITH in capital cities only, thus are potentially conservative.

The number of inpatient reconditioning episodes relied on 2019 data, because of the impact of the COVID-19 pandemic on rehabilitation admissions during 2020—2022 [9]. Further, no adjustment to capital city numbers was made for the small proportion of inpatient episodes involving people in AN-SNAP classes 4AR1 to 4AR4 who were domiciled in residential care prior to the onset of their impairment (< 2%) [4]. While RITH for patients from a residential care facility was outside the scope of this project, the authors are of the view that viable RITH models could be developed for patients returning to residential care for rehabilitation following an acute hospital episode. In this paper we have only presented modelling for the cost of RITH that mirrors an inpatient rehabilitation episode in the public hospital sector. We did not model the cost of the second RITH program delivery model that was supported by Delphi survey participants (i.e. RITH of longer duration and of lesser or variable intensity) [18], but we assume it should be roughly cost equivalent.

## Conclusions

The hypothetical cost of a model of RITH which would provide patients with as comprehensive a rehabilitation service as received in inpatient rehabilitation has been determined, with costing assumptions provided. If programs can be delivered that provide comparable clinical outcomes to those in inpatient rehabilitation, then RITH for the reconditioning impairment category has the potential to assist with Australia’s growing demand for reconditioning following acute hospitalisation, to offer an alternative to inpatient rehabilitation for appropriate patients, and to allow greater system capacity for the same overall expenditure. Future research should focus on trials which compare actual clinical and cost outcomes for RITH, to inpatient rehabilitation.

### Supplementary Information

**Additional file 1.** Methodology for deriving the modelled number of substitutable episodes per annum.

## Data Availability

The data generated and analysed during the current study are available from the corresponding author on reasonable request and pending ethics committee approval.

## Abbreviations

AFRM: Australasian Faculty of Rehabilitation Medicine
ALOS: Average length of stay
AN-SNAP: Australian National Subacute and Non-Acute Patient
AROC: Australasian Rehabilitation Outcomes Centre
FIM: Functional Independence Measure
MDT: Multi-disciplinary team
OOS: Occasion of service
RITH: Rehabilitation in the home
