# Correction: Cost modelling rehabilitation in the home for reconditioning in the Australian context

**DOI:** 10.1186/s12913-024-10759-w

**Published:** 2024-03-20

**Authors:** Roslyn G. Poulos, Andrew M. D. Cole, Dan R. Hilvert, Kerry N. Warner, Steven G. Faux, Tuan‑Anh Nguyen, Friedbert Kohler, Fey‑Ching Un, Tara Alexander, Jacquelin T. Capell, Claire M. C. O’Connor, Christopher J. Poulos

**Affiliations:** 1 HammondCare, Sydney, Australia; 2 Hilvert Advisory, Sydney, Australia; 3 Vincent’s Hospital, Sydney, Australia; 4 South Western Sydney Local Health District, Sydney, Australia; 5 Australasian Rehabilitation Outcomes Centre, University of Wollongong, Wollongong, Australia; 6 School of Psychology, UNSW, Sydney, Australia; 7 School of Population Health, UNSW, Sydney, Australia

**Correction: BMC Health Services Research (2024) 24:151.**

**https://doi.org/10.1186/s12913-023-10527-2**

In Table 3 and 4 of this article, a formatting error occurred in the PDF due to a typesetting mistake. The explanatory text in the final column on the right hand side (Assumptions and Information Sources) in each table has been placed within the first row, rather that running the full length of the table.

The original article has been corrected and the publisher apologises to the authors and readers for the inconvenience caused by these errors.

