# Supplementary material for: Cost modelling rehabilitation in the home for reconditioning in the Australian context
Source: BMC Health Serv Res. 2024 Jan 30;24:151. doi: 10.1186/s12913-023-10527-2 (PMC10826097; doi:10.1186/s12913-023-10527-2)
Supplement: Supplementary file 1 — Additional file 1. Methodology for deriving the modelled number of substitutable episodes per annum. [file 12913_2023_10527_MOESM1_ESM.docx]

Additional File 1

Methodology for deriving the modelled number of substitutable episodes per annum.

|  |  | 4AR1 | 4AR2 | 4AR3 | 4AR4 | Total/  Weighted  average |  | Sources and methodological notes |
| --- | --- | --- | --- | --- | --- | --- | --- | --- |
| Reconditioning episodes in 2019 AROC survey classified 4AR1-4AR4 | A | 8,343 | 10,376 | 3,777 | 1,490 | 23,986 |  | Source^1^: AROC, State of Nations 2019, Reconditioning Episodes in 2019, page 92. 2019 selected (as opposed to a more recent year) because 2019 is pre COVID and therefore more likely to represent future (post COVID) years. |
| Estimated proportion of reconditing beds in AROC survey | B | 98.0% | | | | |  | ^2^AROC data cover approximately 98% of rehabilitation episodes. |
| Number reconditioning episodes in 2019 | C | 8,513 | 10,588 | 3,854 | 1,520 | 24,476 |  | = A / B. This calculation adjusts the AROC survey number to account for the (few) episodes not included in the AROC survey. |
| Proportion of episodes conducted in public hospital facilities, 2019 | D | 31.0% | 32.7% | 48.2% | 53.8% | 35.9% |  | Source^1^: AROC, State of Nations 2019, Reconditioning Episodes in 2019, page 92. |
| Number reconditioning episodes in 2019 in public hospital facilities | E | 2,639 | 3,462 | 1,858 | 818 | 8,777 |  | = C * D. |
| Estimated proportion of episodes amenable to at-home substitution | G | 49.7% | 51.8% | 20.7% | 21.0% | 41.7% |  | Estimated potential utilisation of RITH by AN-SNAP class (n=21 participants). See Table 2. |
| Proportion of patients living in state capital cities | H | 67.2% | | | | |  | Source^3^: ABS Regional Population Series, 2021, Table 4, shows 2021 capital cities population = 17,252,876 and non-capital city population = 8,435,203. |
| Proportion of inpatient episodes in capital cities and amenable to at-home setting | I | 33.4% | 34.8% | 13.9% | 14.1% | 28.0% |  | = G * H. |
| Number of substitutable episodes per annum | J | 881 | 1,205 | 258 | 115 | 2,459 |  | = E * I. This is the number of reconditioning episodes conducted in public hospital facilities but discounted to count only the proportion of patients amenable to home substitution and then further discounted to count only patients living in state capital cities. |

Sources:

1. Australasian Rehabilitation Outcomes Centre (AROC). AROC Annual Report – The state of inpatient rehabilitation in Australia in 2019 Australasian Rehabilitation Outcomes Centre, Australian Health Services Research Institute, University of Wollongong; 2020. Available from: https://ro.uow.edu.au/cgi/viewcontent.cgi?article= 2131&context=ahsri
2. Australasian Rehabilitation Outcomes Centre (AROC). Australian Clinical Quality, Registries Project for ACS&QHC. Final Report 2009. Available from: https://www.safetyandquality.gov.au/sites/default/fles/ migrated/21_Australasian-Rehabilitation-Outcomes-Centre-AROC- REPORT-PDF-504-KB.pdf
3. Australian Bureau of Statistics. Regional population 2021. Available from: https://www.abs.gov.au/statistics/people/population/regional-population/2021 (accessed 28 June 2022)
